# The relationship between obesity and prostate cancer: from genetics to disease treatment and prevention

**DOI:** 10.1186/1741-7015-10-109

**Published:** 2012-09-25

**Authors:** Giovanni Lughezzani

**Affiliations:** 1 Department of Urology, Vita-Salute San Raffaele University, Milan, Italy

**Keywords:** Prostate cancer, obesity, adipose tissue, gene expression

## Abstract

Recent studies demonstrated that obesity is associated with prostate cancer aggressiveness and prognosis. However, the mechanisms underlying this relationship are poorly understood. Tumor microenvironment has been increasingly considered as an important determinant of cancer growth and progression. In the light of this growing evidence, Ribeiro *et al*., in a *BMC Medicine *research article, investigated the gene expression profiles of periprostatic adipose tissue of obese patients with and without prostate cancer and compared them to those of lean patients. Their findings provide the first evidence of a differential gene expression in the periprostatic adipose tissue of obese individuals. Differences were also observed when comparing the periprostatic adipose tissue of patients with and without prostate cancer. Differentially expressed genes are related to cell proliferation and immunological responses. Besides suggesting the genetic bases for the observed relationship between obesity and prostate cancer aggressiveness, these findings provide new insights on the important link between local microenvironment and cancer progression.

## Background

Obesity represents a well-recognized risk factor in several malignancies, such as gastroenteric tract cancers. Conversely, the relationship between prostate cancer and obesity is still a matter of debate. Recent studies showed that obesity is related to higher prostate cancer grade and stage, as well as to worse oncological outcomes [1-4].

Emerging data suggest the importance of the interactions between cells in the microenvironment and tumor cells to determine cancer growth and progression [5]. When focusing on the prostate, periprostatic (PP) adipose tissue represents the first structure outside the organ capsule; its infiltration by tumor cells has a detrimental effect on the prognosis of patients with prostate cancer [6]. To date, only a few studies investigated the interactions between the adipose microenvironment and prostate cancer cells [7-9]. These studies concluded that PP adipose tissue may play an important role by releasing cytokines and growth factors, such as interleukin-6 and matrix metalloproteinases, that may promote tumor cell proliferation and migration. These interactions may have a key-role in determining prostate cancer aggressiveness and progression.

However, to date, no studies have investigated whether the relationship between prostate cancer and obesity may be attributable to changes in the genetic characteristics of PP adipose tissue of obese individuals. Ribeiro *et al*. [10] have attempted to answer this question by determining the gene expression profiles of PP adipose tissue of obese individuals and comparing them with those of lean patients with or without prostate cancer.

### Obesity and prostate cancer: unraveling the mechanisms behind the association

While growing evidence suggests an important link between obesity and several human malignancies, the mechanisms underlying this relationship are still poorly understood. In the current study, evaluation of the PP adipose tissue of 18 patients (6 with benign prostatic hyperplasia, 6 with organ-confined prostate cancer and 6 with extra-prostatic prostate cancer) was performed. Tissue samples were collected during surgery. In each group, three patients were lean and three patients were obese/overweight. The authors identified several genes differentially expressed in the PP adipose tissue of obese patients (for example, FADS1, LEP and ANGPT1). These genes are mainly related to anti-lipolytic, lipogenic, proliferative and anti-apoptotic activities. Genes linked to the inflammatory response (for example, NPY1R and FADS1), were also differentially expressed in the PP adipose tissue of obese patients, thus determining a favorable environment for disease progression.

Similarly, the authors showed that several genes involved in cell cycle and proliferation, as well as those involved in adipocytes differentiation (for example, PLCB1 and FFAR2) were differentially expressed in the PP adipose tissue of prostate cancer patients, determining an increase in its thickness. These findings are particularly interesting in light of a recent study that showed a relationship between PP adipose tissue thickness and prostate cancer aggressiveness [11]. In addition, up-regulation of genes that reduce immunosurveillance, which may favor prostate cancer progression, was also observed.

Taken together, these findings provide new insights into the importance of PP adipose tissue in creating a favorable environment for prostate cancer cells proliferation and disease progression.

To date, only a few studies have examined the link between PP adipose tissue and prostate cancer. Finley *et al*. observed an increase in interleukin-6 (IL-6) in the PP adipose tissue of prostate cancer patients relative to individuals without prostate cancer [7]. In addition, IL-6 levels correlated with tumor grade [7]. Similarly, Ribeiro *et al*. showed that the PP adipose tissue may modulate prostate cancer cells' growth and migration through a local increased activity of matrix metalloproteinase (MMP) [8]. Finally, Sacca *et al*. confirmed these observations by demonstrating an increased secretion of pro-MMP-9 by the PP adipose tissue of patients with PCa [9]. These findings confirmed the fundamental role of local microenvironment in determining cancer cells proliferation and migration.

However, the mechanisms regulating the secretion of cytokines and growth factors by PP adipose tissue are still largely unknown. In the current study, several genes differentially expressed both by the PP adipose tissue of obese patients and by the PP adipose tissue of prostate cancer patients were identified, suggesting that pathways involved in cell growth and proliferation, as well as in immunological responses, may be altered in these individuals. Based on their findings, the authors hypothesize a "cross-talk" mechanism between PP adipose tissue and cancer cells that may ultimately result in more aggressive prostate cancer and promote disease progression, especially in obese patients.

While this hypothesis should be interpreted with caution due to the small number of patients included in the current preliminary study, these findings provide important evidence regarding the key role of the adipose microenvironment in determining prostate cancer aggressiveness and tumor progression, and shed light on the genetic pathways that may be involved in the complex relationship between obesity and prostate cancer.

### Future directions and conclusions

The differential expression of several genes represents the key for identifying pathways involved with cancer cell proliferation and tumor progression. In this light, the importance of the current study is at least two-fold. First, Ribeiro *et al*. demonstrated a different gene expression in obese patients relative to lean patients, irrespective of the presence of prostate cancer. By showing an up-regulation of genes related to lypolitic, lipogenic, proliferative and anti-apoptotic activities in obese patients, this study provides the first genetic explanations for the observed relationship between obesity and prostate cancer aggressiveness. Second, the authors demonstrated different gene signatures in the PP adipose tissue of prostate cancer patients, suggesting the presence of a "cross-talk" mechanism between tumor cells and adipose tissue cells to create a favorable environment to prostate cancer progression.

These findings should promote future studies to further investigate the relationship between local microenvironment and cancer cell growth. The genetic pathways that were identified in the current study may represent potential objectives for targeted therapies. In addition, gene expression profiles may also be used to better stratify the prognosis of patients with prostate cancer. Finally, the better understanding of the PP adipose tissue biology may further promote the development of chemo-preventive strategies and lifestyle measures meant to reduce the number of life-threatening prostate cancers.

## Abbreviations

IL-: interleukin 6; MMP: matrix metalloproteinase; PP: periprostatic

## Competing interests

The author declares that they have no competing interests.

## Author's information

GL completed a post-doctoral fellowship in urologic oncology at the Cancer Prognostics Health Outcomes Unit in Montreal. He currently works at the Department of Urology of Vita-Salute San Raffaele University in Milan. His research interests focus mainly on prostate, kidney and urothelial carcinoma.

## Pre-publication history

The pre-publication history for this paper can be accessed here:

http://www.biomedcentral.com/1741-7015/10/109/prepub
